# Colorectal Cancer in Northern Tanzania: Increasing Trends and Late Presentation Present Major Challenges

**DOI:** 10.1200/JGO.19.00301

**Published:** 2020-03-03

**Authors:** Ayesiga M. Herman, Alexander T. Hawkins, Kennedy Misso, Christian Issangya, Murad Tarmohamed, Alex Mremi, Furaha Serventi, David Msuya, Rune Philemon

**Affiliations:** ^1^ Department of General Surgery, Kilimanjaro Christian Medical Center, Moshi, Tanzania; ^2^ Kilimanjaro Christian Medical University College, Moshi, Tanzania; ^3^ Division of General Surgery, Colon and Rectal Surgery, Vanderbilt University Medical Center, Nashville, TN; ^4^ Department of Pathology, Kilimanjaro Christian Medical Center, Moshi, Tanzania; ^5^ Department of Oncology, Kilimanjaro Christian Medical Center, Moshi, Tanzania

## Abstract

**PURPOSE:**

A trend of increasing incidence of colorectal cancer (CRC) has been observed in northern Tanzania. Studies have shown a six-fold increase in CRC in the past decade, with 90% of patients presenting in late stages, with resultant high morbidity and mortality rates. In this study, we aimed to document the burden of CRC in the northern zone of Tanzania from 1998 to 2018, focusing on patient presentation, clinical features, and treatment at a tertiary hospital.

**METHODS:**

Pathological and clinical records for all patients from 1998 to 2018 were identified and reviewed. Records of patients whose CRC was diagnosed histologically were retrospectively reviewed.

**RESULTS:**

Approximately 313 CRC cases were documented. The majority age group (29.1%) was between 50 and 64 years (mean [standard deviation], 54.28 [16.75] years). However, together, the age groups of patients younger than 50 years was 41.5% (n = 130). Of 174 patients with complete records, most (29.3%) were between 35 and 49 years of age. The median age was 52 (interquartile range, 40-67) years. Men accounted for 62.1% of patients and were mostly from the Kilimanjaro region. More than half (54.7%) presented > 3 months after symptom debut; 62.6% first sought care at lower-level health facilities. Most (64.9%) presented as emergencies, necessitating colostomy for fecal diversion as the initial surgical procedure in 60.3% of patients. Colonoscopy was performed for 38.6% of the study participants. Most tumors (72.7%) originated from the sigmoid and rectum. Adenocarcinoma was the most prevalent histologic type.

**CONCLUSION:**

High proportions of young individuals with CRC pose great concern and a need for further appraisal. Furthermore, late emergency presentation and low colonoscopy rates highlights underlying system challenges and need for education campaigns.

## INTRODUCTION

Colorectal cancer (CRC) is common worldwide, and incidence varies around the globe.^1^ It is ranked the fourth most common cancer in men after prostate cancer, lung cancer, and gastric cancer, and third in women after breast and cervical cancers.^2,3^ CRC contributes to significant morbidity, and mortality rates are reported to be on the increase in low-resource settings.^1^ However, CRC has good prognosis when managed well in early stages, with a 5-year survival rate of up to 90%.^4^

In the past years, there was low incidence of CRC in developing countries.^1^ However, a change in this trend has been documented, whereby more cases are being reported.^5^ For sub-Saharan countries, reviews done in the year 2012 and 2017 reported an increasing incidence.^5,6^ However, some authors have reported difficulties in estimating the incidence because of poor epidemiologic data.^5^ Lack of surveillance data and tracking mechanisms in most African countries make it difficult to estimate the burden of CRC.^7^

In Tanzania, a six-fold increase in CRC incidence has been documented in the past decade, with the incidence in the Kilimanjaro region estimated at 4.4 per 100,000 population—the third highest in the country after Dar-es-Salaam and the Pwani region.^8^ With our communities rapidly moving toward urbanization, alteration in diet and inactivity are among the major risk factors attributed to this observation. However, together with these behavioral changes, there are other unexplored genetic, familial factors and infectious causes whose contribution to the development of CRC in our population is yet to be fully investigated.^9^ Exploring these factors in our communities may help address the increasing incidence and the observed trend of young individuals being affected by the disease.^5,8,10^

**Context**

**Key Objective**
  To document the burden and describe presentation and distribution of colorectal cancer (CRC) in northern Tanzania.
**Knowledge Generated**
The high proportion of individuals younger than 50 years of age who are affected by CRC is comparable to other data reported in studies performed in African countries. Late presentation, mostly under emergency bases, was a common phenomenon among the study participants. Rates of colonoscopy are low, mostly because of limited accessibility to services and a lack of screening programs for CRC in the country.
**Relevance**
We call for strengthening the health system in effective screening and education campaigns to help with early detection and robust surveillance.

We conducted a record review from 1998 to 2018 for patients with CRC in our center, with the aim of describing the burden of the disease, clinical characteristics of patients, and the extent of the disease at diagnosis. We describe the histologic morphology and grading of CRC, as well as treatment offered patients with CRC who attended the main tertiary hospital in Northern Tanzania.

## MATERIAL AND METHODS

We conducted a retrospective review of records for all patients histopathologically diagnosed with CRCs and who attended our hospital from 1998 to 2018; our aim was to describe their clinical characteristics and treatment offered. The record search was done from the pathology laboratory, followed by retrieval of the patient’s clinical data in the medical records. Patients with important missing information were excluded from the study. Tissue for histopathology was obtained either through endoscopy (colonoscopy), or during surgery as resection or biopsy during stoma diversion for most patients.

Initial baseline evaluation of affected individuals, demographic data, presenting symptoms and their duration, extent of disease at diagnosis, mode of presentation, and initial treatment offered were documented. Pathologic grading and type were also documented.

The extent of disease (severity) was categorized as localized (ie, involving colon or rectum), regional disease (ie, involving lymph nodes, pelvic wall, or adjacent organs), or distant spread for metastasis to distant or adjacent organs.^7,11^

Data analysis was done using SPSS, version 23 (IBM, Armonk, NY). Numeric variables were summarized using measures of central tendency with their respective measures of dispersion. Categorical variables were summarized using frequencies and percentages. The study received approval from the Kilimanjaro Christian Medical University College ethical committee (approval no. 2077).

## RESULTS

**FIG 1:**
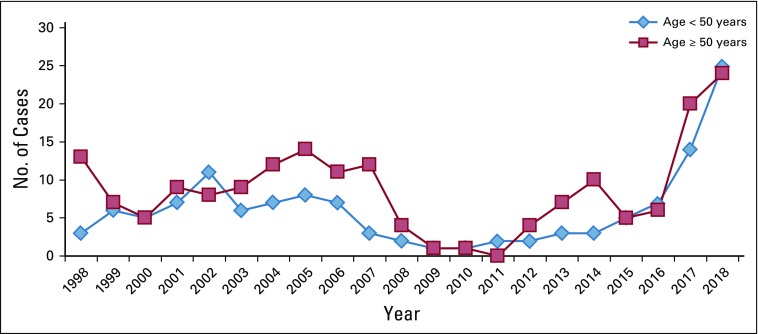
Trend in colorectal cancer (CRC) cases (absolute numbers per year) from 1998 to 2018, based on the age categories < 50 years and ≥ 50 years (N = 313). The mean age at diagnosis was 54.28 years (standard deviation, 16.75).

Between 1998 and 2018, a total of 313 CRC cases were documented in the pathology registry (Fig 1). By age group, the majority of patients (29.1%) were between 50 and 64 years of age (mean age [standard deviation], 54.28 [16.75] years). Together, patients in age groups younger than 50 years accounted for 41.5% (n = 130) of the cohort. Male sex predominated, with a male-to-female ratio of 1.3:1.

Of 313 documented cases, important information was missing for 139 patients with CRC and these were excluded from the rest of the descriptive statistics analyses. For the 174 patients who were included in the analysis, most (29.3%) were between the ages of 35 and 49 years; the median age was 52 (interquartile range, 40-67) years. Male patients made up almost two-thirds of this cohort; the male-to-female ratio was 1.6:1. Most of the patients were from the Kilimanjaro region (Table 1).

**TABLE 1:**
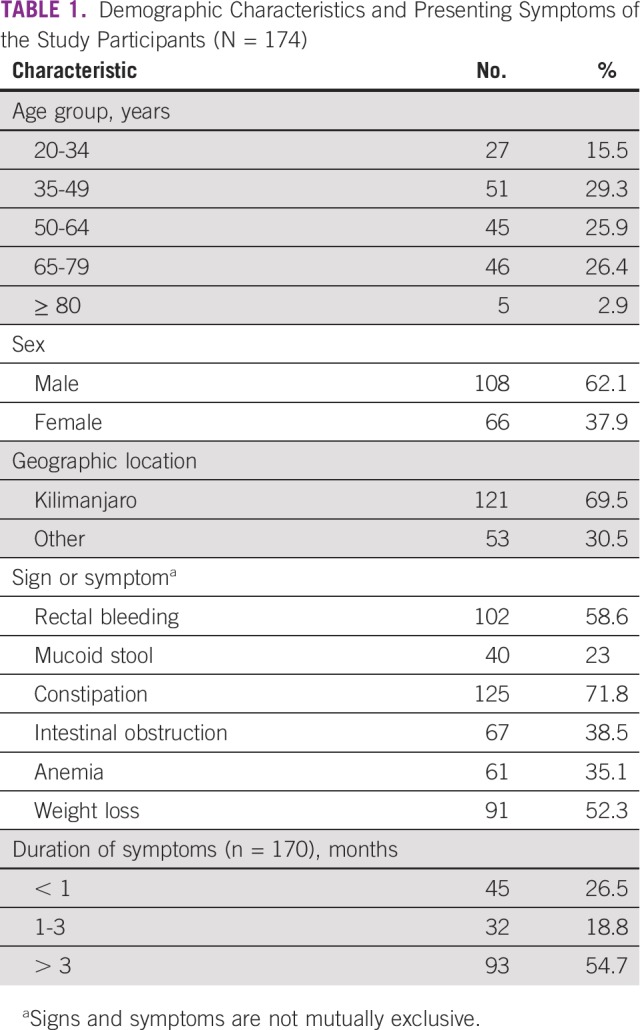
Demographic Characteristics and Presenting Symptoms of the Study Participants (N = 174)

The presenting symptoms included constipation (71.8%), weight loss (52%), rectal bleeding (58.6%), bowel obstruction (38.5%), anemia (35%), and mucous stool in 23% of all study participants. Approximately 54.7% of all patients presented > 3 months after becoming aware of their symptoms (Table 1)

**FIG 2:**
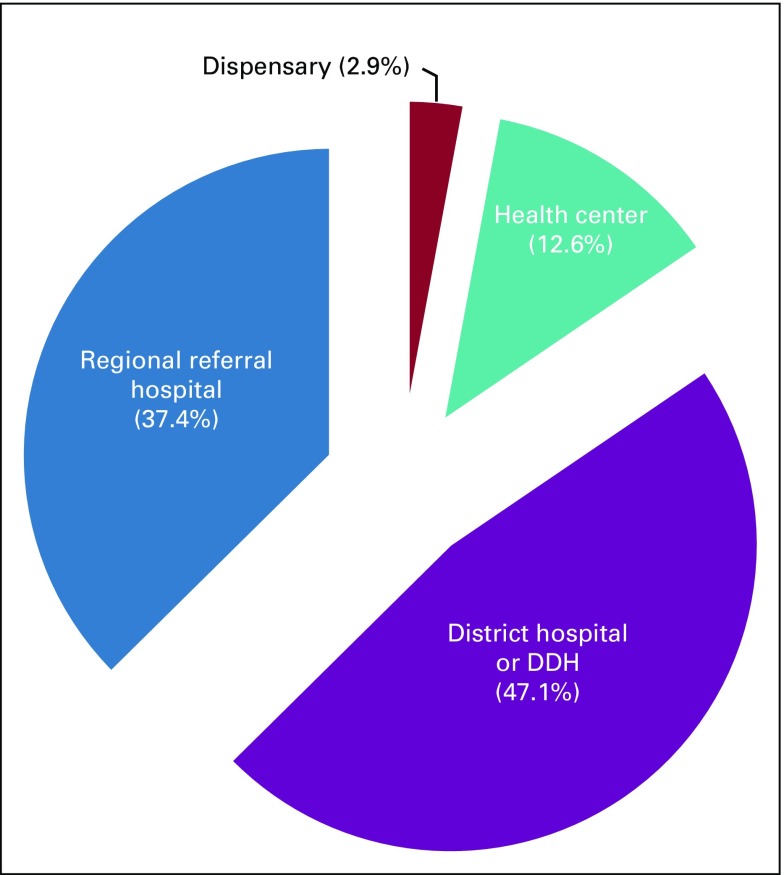
Levels of health facilities and percentage of patients attended at those facilities on recognition of colorectal cancer symptoms. Approximately one-half (47.1%) of patients were initially attended at district hospitals or designated district hospitals (DDH).

Most patients (62.6%) were seen at a district hospital, health center, or dispensary for their initial evaluation after becoming aware of their symptoms (Fig 2). Most patients (63.2%) were referred to our tertiary center from other health facilities, and almost two-thirds presented on an emergency basis (Table 2).

**TABLE 2:**
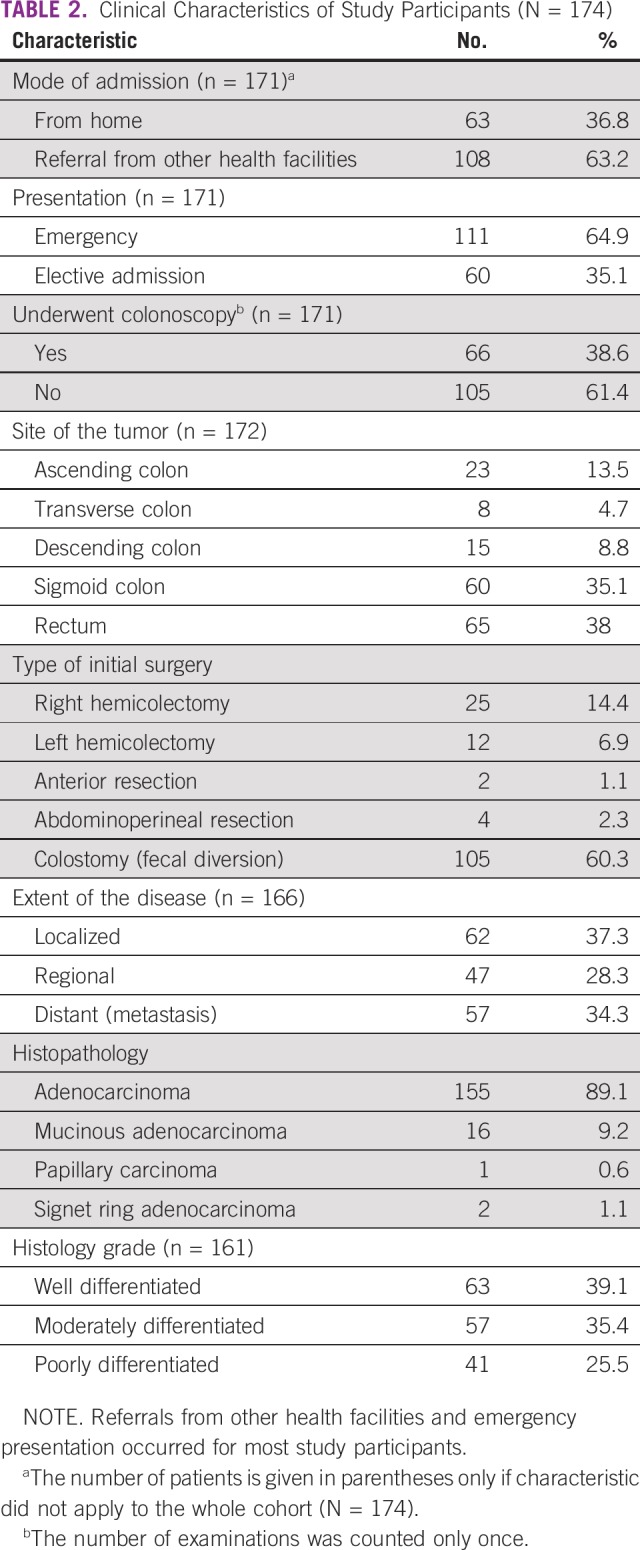
Clinical Characteristics of Study Participants (N = 174)

Colonoscopy was performed in only 38.6% of the study participants. Computed tomography scan was done in 67 of the 174 patients. Approximately three-quarters of the tumors originated from the sigmoid colon and the rectum. Colostomy for fecal diversion was performed in 60.3% of the patients as their initial surgical procedure. Approximately 37.3% of the patients had localized disease to the colon or rectum; distant disease (ie, metastasis) was found in 34.3% of the patients. Adenocarcinoma was the most prevalent tumor type according to histology. Histologic grade was classified as well-differentiated for approximately 40% of tumors, followed by moderately differentiated (35.4%) and poorly differentiated (25%) tumors (Table 2).

## DISCUSSION

With our rapidly changing communities in sub-Saharan Africa, we are observing changing epidemiology of diseases. There is a documented, significant shift indicating that the incidence of noncommunicable diseases, cancer included, is on the rise.^12,13^ From the records observed at our center, the trend in the incidence of documented CRC cases has been increasing since 2015 (Fig 1). These trends present a major challenge to the health care system in our country. In one study, the Kilimanjaro region was reported to be among the top three regions in the country with high CRC incidence.^8^ With the already strained health care budget and underprepared health systems, it is evident that more work needs to be done in putting forth important infrastructure to combat the burden of CRC in the region.

We postulate the observed trends may partly be due to underreporting of the cases in the past, following irregularities in health care infrastructure. This has been observed and discussed by scholars in the other areas.^14^ Inadequate infrastructure of the health care system hampering accessibility and diagnosis affects most of the developing world. Lack of knowledge and awareness (from the patient’s perspective) of CRC is another major factor that makes patients opt for alternative pathways of treatment once suspicious symptoms emerge.^15^

In recent years, there have been developments in the health care infrastructure in our area since oncologic services were established in December 2016.^16^ This has helped create awareness of cancer in the community, enabling them to access the available services.^16^ Although the coverage is still low, the explanation is plausible, but probably only partly, for clarifying the observed increase in the reporting of CRC cases.

There are three important observations made from the current findings. First is the alarmingly high number of young individuals who are affected by the disease; second is the late emergency presentation of patients; and third is the low rate of colonoscopies (screening and diagnostic) performed for patients, according to the study findings.

With regard to early-onset CRC, CRC in individuals of African origin has been observed to develop at a relatively younger age^5,10,17,18^ compared with white people.^19,20^ Arguments on the difference in tumor biology and risk factors that place individuals of African descent at a relatively higher risk have been pointed out.^9,21^ Some of the hypotheses made include association of CRC with infectious agents, including the contribution of colonic microbiota and genetic predisposition or familial factors.^9^ These factors call for more research to explore the etiopathogenesis of CRC in African settings. Most of the screening guidelines we use in Africa are adopted from western countries, whereby the recommended age for initial screening for CRC in an average-risk patient is 50 years.^22,23,24^ A crucial message from our observations in this study is that these criteria will potentially miss approximately 40% of the individuals affected by the disease in our setting. This calls for development of guidelines that suit our populations and suggests the factors that play roles in CRC development in our area may be different, demanding different approaches for screening and early detection. Some studies done in United States after the increase in incidence of early-onset CRC have suggested lowering the screening age for individuals of African descent.^22,25^

The second observation is on low rates of colonoscopy in our study participants. Only 38% of individuals in the current study had ever had a colonoscopy performed for screening or diagnostic purposes. These rates were similar for individuals who were younger than or older than 50 years (37.7% and 38.8%, respectively). In other studies, the colonoscopy rates differ because most of the patients had health care access either during surveillance or screening programs.^26^ In our settings, establishing these facilities have a huge cost implication, and they are mostly accessible at the regional referral health facilities where, from our record review, only 37.4% of individuals were seen initially by a health care provider. Lack of community sensitization to screening and scarcity of equipment and skilled personnel to perform colonoscopies are among the factors contributing to low colonoscopy rates. Most of the patients whose records were used in this review were first attended at dispensaries, health centers, and district hospitals. Few of these health facilities have infrastructure for endoscopic procedures. Providing these hospitals with trained people, at least at the district levels, and investigative capabilities, which are cost effective, together with implementing tailored screening programs may have an important impact on surveillance and early detection of the CRCs. Such developments require collaboration at local, regional, and national levels.

Third, there is a general lack of awareness in our community about CRC and, importantly, a lack of screening programs. Numerous campaigns in Tanzania have been done on breast cancer^27^ and cervical cancer,^28^ mobilizing and encouraging the community to screen and to come early once they notice suspicious symptoms. However, the situation is different for CRC. Recognizing the need, Kilimanjaro Christian Medical Center, through its oncology unit, has conducted successful campaigns for all types of cancers, including CRC, since 2017 throughout the northern zone of Tanzania.^29^ These kinds of activities need to be rolled out to most parts of the country to raise awareness and must go hand in hand with education of health care personnel, particularly in the peripheral health centers, on identification and appropriate and timely diagnosis for patients with suspicious symptoms. With the findings of emergency presentations in most of the patients in our review, sensitizing the community to screening and early recognition of suspicious symptoms is of paramount importance.

### Limitations

This study has a number of limitations with regard to case identification, missing patient information, and lost follow-up. For case identification, we used the pathology registry because of its reliability in capturing most of the patients with CRC who attended our center during the study period. However, with this approach, it is highly likely that we missed any clinically diagnosed CRC tumor that did not undergo biopsy or resection.

Second, many patients were not included in the study because of missing information. However, from the few data that were obtained, we think this group of patients was comparable in age and sex to the 174 patients who were analyzed. Some reasons for the missing patients include the following: (1) Because we established our study participants from the pathology registry, samples sent from other hospitals for histopathology were unlikely to have a reference number in the medical records, even though the occurrence of CRC was documented. (2) Some files were missing information from the patient’s clinical presentation and operative findings, thus these were difficult to characterize beyond the documented occurrence of the disease. (3) We think some files might have been misplaced with the transfer of patients to other centers for oncologic services.

Third, because of patients lost to follow-up and the previous protocol of referral to a different hospital for oncologic services, obtaining survival data was not possible.

To conclude, CRC poses a significant burden on the community in our setting, with notably increasing trends that are only partially explained by improving health services and diagnoses. The high number of young individuals affected is a great concern and there is a need to re-examine the effectiveness of traditional screening in those > 50 years of age. System challenges have been highlighted, with poor recognition of symptoms by the health care workers and patient factors attributed to lack of education. All these lead to late presentation on an emergency basis and affect morbidity and mortality. The way forward is strengthening of the health system with effective screening and education campaigns. These must be coupled with research into effective and age-appropriate specific screening methods for our setting, together with additional research exploring molecular etiologies of these cancers and their link to infections prevalent in the region.
